# Comparative performance of four large language models in generating evidence-based exercise prescriptions using FITT-VP framework

**DOI:** 10.3389/fphys.2026.1846567

**Published:** 2026-05-25

**Authors:** Huan Feng, Xiaojun Wang

**Affiliations:** College of Physical Education, Sichuan University, Chengdu, China

**Keywords:** artificial intelligence, claude 3.7, clinical decision support, comparative evaluation, DeepSeek R1, exercise prescription, FITT-VP framework, GPT-4o

## Abstract

**Background:**

Exercise prescription plays a critical role in health management, but effective implementation is limited by practitioner expertise and time constraints. Large language models (LLMs) offer potential for generating personalized prescriptions, yet their comparative performance within established frameworks remains unexplored.

**Methods:**

This study evaluated four advanced LLMs (GPT-4o, Claude 3.7, DeepSeek R1, and Grok-3) in generating exercise prescriptions based on the FITT-VP framework (Frequency, Intensity, Time, Type, Volume, Progression). Thirty synthetic patient profiles were designed from epidemiological data and clinical guidelines, and validated through a three-stage process involving sports medicine students and expert review. Three certified exercise specialists independently rated each prescription across the six FITT-VP dimensions using a 0–10 scale. One-way repeated measures ANOVA with Bonferroni correction and effect size calculations were applied.

**Results:**

Across six FITT-VP dimensions (maximum total: 60), Claude 3.7 showed the highest total score (50.23 ± 1.75), followed by Grok-3 (47.42 ± 1.50), GPT-4o (44.02 ± 1.68), and DeepSeek R1 (40.30 ± 1.46). ANOVA revealed significant differences among models (F = 250.58, p < 0.001, η² = 0.896). *Post-hoc* analysis confirmed significant differences between Claude 3.7 and both GPT-4o (p < 0.001, d = 2.83) and DeepSeek R1 (p < 0.001, d = 5.01). Claude 3.7 excelled in Time (8.69 ± 0.65) and Progression (8.46 ± 0.96), while DeepSeek R1 was weakest in Intensity (6.59) and Type (6.65). Grok-3 performed competitively in Volume but struggled with complex comorbidities.

**Conclusion:**

This study establishes important benchmarks for AI in exercise medicine, with Claude 3.7 showing promise as a drafting tool for individualized exercise prescriptions and supporting a collaborative human–AI framework. These results, however, are based on a single-run evaluation of static synthetic profiles and expert-rated prescription quality rather than real-world clinical outcomes; multi-run variability assessment and clinical validation with human–AI interaction and patient follow-up are required before implementation.

## 1 Introduction

Exercise prescription plays a pivotal role in contemporary healthcare management, serving as a structured approach to delivering personalized physical activity recommendations for health promotion, disease prevention, and therapeutic interventions (Lobelo et al., 2014; Pedersen and Saltin, 2015). As defined by the American College of Sports Medicine (ACSM), exercise prescription encompasses the systematic process of designing and implementing individualized exercise programs based on scientific principles and tailored to a person’s health status, fitness level, and goals (Arad et al., 2015). This evidence-based framework has evolved substantially over recent decades, transitioning from generalized recommendations to precision-based protocols that consider multiple physiological, psychological, and environmental factors (Piercy et al., 2018; Ross et al., 2019).

The scientific foundation of exercise prescription rests on well-established physiological principles that govern the human body’s response to physical activity (Hawley et al., 2014). Research has consistently demonstrated that these adaptations can be manipulated through systematic variation of exercise parameters—frequency, intensity, time, type, volume, and progression (FITT-VP)—to achieve specific health outcomes (Garber et al., 2011; Egan and Zierath, 2013). The FITT-VP framework has emerged as the gold standard for structuring exercise prescriptions, providing clinicians with a systematic approach based on comprehensive guidelines published by authoritative bodies such as the ACSM, the World Health Organization (WHO), and various specialty medical associations (Wasfy and Baggish, 2016; Eijsvogels et al., 2018). These guidelines emphasize pre-exercise screening, establishing measurable goals, selecting appropriate exercise modalities, and implementing progressive overload principles with regular reassessment to optimize outcomes and adherence (Riebe et al., 2015; Bull et al., 2020).

Despite these advances, significant challenges persist in current exercise prescription practices. Clinician-related barriers include insufficient training in exercise science, time constraints during consultations, and lack of confidence in prescribing exercise for complex medical conditions (Lobelo et al., 2009; Stoutenberg et al., 2017). Patient-related challenges encompass poor adherence, inadequate health literacy, and socioeconomic constraints that limit access to exercise facilities (Rhodes et al., 2017). Systematic limitations include difficulty translating population-based guidelines to individualized prescriptions and inadequate integration into electronic health records (Joy et al., 2013; Cooper et al., 2021). The traditional one-size-fits-all approach fails to account for individual genetic variations, personal preferences, and cultural factors that influence exercise response and adherence (Buford et al., 2013).

The emergence of large language models (LLMs) represents a potentially transformative development in healthcare communication, including the generation of medical texts such as exercise prescriptions (Kung et al., 2023). These sophisticated artificial intelligence systems, trained on vast corpora of text, demonstrate remarkable capabilities in generating coherent, contextually appropriate, and stylistically diverse content (Thirunavukarasu et al., 2023). Recent evaluations of LLMs in medical text generation have assessed various performance metrics, including factual accuracy, clinical relevance, and adherence to established guidelines (Singhal et al., 2023; Foster et al., 2025). Studies by Thirunavukarasu et al. (Goh et al., 2023) and Ayers et al. (2023) found that advanced LLMs could generate medical recommendations that were often indistinguishable from those written by healthcare professionals, though concerns about occasional factual errors and hallucinations persist. The accuracy and reliability of AI-assisted medical decision support systems, including those powered by LLMs, remain active areas of investigation (Davenport and Kalakota, 2019). Research indicates that while these systems show promising performance in structured tasks with clear guidelines—such as generating exercise prescriptions based on established protocols—their performance may be less consistent in complex scenarios requiring nuanced clinical judgment (Rajkomar et al., 2019). A systematic review by Johnson et al. (2021) found that AI systems achieved an average accuracy of 87% across various medical decision-support tasks, though performance varied significantly by medical specialty and task complexity. Concerns regarding reliability center on issues such as algorithm transparency, training data biases, and the potential for outdated information if models are not regularly updated with the latest medical evidence (Gerke et al., 2020). Additionally, the non-deterministic nature of LLM outputs presents challenges for reproducibility, as these systems may generate different responses when presented with identical inputs under varying conditions (Ayers et al., 2023).

The integration of AI-powered tools in exercise prescription represents a promising frontier for addressing these current limitations while enhancing personalization capabilities (Silva et al., 2022). By leveraging computational power to analyze multiple patient variables simultaneously, AI systems can potentially generate more precisely tailored exercise recommendations than traditional methods (Friedrich et al., 2021). Early studies suggest that AI-assisted exercise prescription may improve patient satisfaction, adherence rates, and clinical outcomes compared to conventional approaches (Fonseca et al., 2018; Hall et al., 2022). However, the comparative performance of different AI models in generating exercise prescriptions—particularly with respect to accuracy, guideline adherence, individualization, and alignment with established frameworks like FITT-VP—remains largely unexplored (Maron et al., 2018).

The present study aims to address this research gap by systematically comparing the performance of four advanced large language models (GPT-4o, Claude 3.7, DeepSeek R1, and Grok-3) in generating exercise prescriptions based on the FITT-VP framework. By evaluating these models across multiple dimensions of exercise prescription quality, this research seeks to provide insights into the current capabilities, limitations, and potential applications of AI in exercise medicine. The findings may inform future developments in AI-assisted clinical decision support systems while contributing to the broader understanding of how these emerging technologies can complement healthcare professionals in delivering optimal, personalized exercise recommendations for diverse patient populations.

## 2 Methodology

### 2.1 Research design

This study employed a comparative research framework to evaluate the performance of four large language models (LLMs)—GPT-4o, Claude 3.7, DeepSeek R1, and Grok-3—in generating exercise prescriptions based on the FITT-VP principle. The comparative design was selected for its established utility in identifying relative strengths and limitations across multiple interventions or technologies when evaluated against standardized criteria (Bryson et al., 2012). As noted by Whittaker et al. (2018), comparative designs are particularly valuable in the rapidly evolving field of artificial intelligence, where benchmarking against established standards can provide meaningful insights into model capabilities and limitations. Regarding the 30 patient profiles utilized in this study, it is important to note that these were synthetic profiles created based on epidemiological data (Walonoski et al., 2018) and clinical guidelines rather than data collected directly from actual patients. The profiles were developed through a rigorous three-stage validation process involving sports medicine students and expert review (Chen et al., 2019) to ensure clinical realism and representativeness. This approach eliminated the need for direct patient recruitment while still maintaining clinical relevance. No actual patient data was collected or used in this research (Blandford et al., 2020), Three certified exercise specialists who served as expert raters provided informed consent prior to their participation as evaluators. This methodological choice allowed for standardized testing across all models while protecting patient privacy and adhering to ethical research practices (Rankin et al., 2020).

### 2.2 Sample size

A systematic evaluation protocol was developed following the methodological framework proposed by Sounderajah et al. (2021) for assessing AI applications in healthcare. The study utilized a cross-sectional design with repeated measures, wherein each LLM was presented with identical patient profiles and prompt structures to generate exercise prescriptions, allowing for direct comparison of outputs across models under controlled conditions (Schulz et al., 2010).

The sample size determination of 30 patient profiles was based on statistical power considerations and practical feasibility constraints. Sample size calculations were performed using G*Power 3.1.9.7 software (**Supplementary Figure 1**). Based on Cohen’s power analysis guidelines for repeated measures designs (Cohen, 2013), with an expected effect size of f = 0.25 (equivalent to Cohen’s d = 0.5, consistent with AI healthcare evaluation studies (Rajput et al., 2023; Tan et al., 2024) and empirical observations in exercise prescription research (Doherty et al., 2024)), alpha level of 0.05, power of 0.8, correlation among repeated measures of 0.5 (based on established patterns in repeated measures healthcare studies (Bakdash and Marusich, 2017; Muhammad, 2023)) and 4 repeated measures (models), the minimum required sample size was calculated as 24 patient profiles. We used 30 patient profiles to ensure adequate power (actual power = 0.95) and provide a safety margin for potential data variability. The power analysis refers to the number of distinct clinical scenarios evaluated, not to human participants. This sample size provided 120 total assessments (30 profiles × 4 models), ensuring sufficient statistical power for detecting meaningful differences between models. Additionally, the sample size determination was informed by previous benchmarking studies of LLMs in healthcare applications, which demonstrated that reliable performance patterns could be established with 25–30 distinct clinical scenarios (Dave et al., 2023; Lee et al., 2023).

### 2.3 Patient profile design

The 30 patient profiles were strategically designed to represent diverse demographics, health conditions, and exercise backgrounds. Target distributions for key variables were anchored to published real-world epidemiological data rather than defined *ad hoc*. Age structure, BMI category proportions, and the prevalence ranges of major chronic conditions (type 2 diabetes, hypertension, cardiovascular disease) were informed by NHANES 2017–March 2020 prepandemic estimates and the China Chronic Disease and Risk Factor Surveillance 2018 survey (Stierman et al., 2021; Zhang et al., 2023); exercise-related variables followed ACSM pre-participation screening recommendations. The prevalence of type 2 diabetes, multiple comorbidities, and sedentary behaviour was intentionally set above general-population estimates to enable stress-testing of model performance on clinically demanding cases, a choice discussed in the Limitations. A benchmark comparison against the reference sources is provided in **Supplementary Table 4**. No real patient data were collected or analysed; all profiles are fully synthetic and are not a substitute for clinical validation in actual patients. The complete characteristics of the synthetic patient profiles are presented in **Supplementary Table 3**. Profiles spanned ages 20–75 years with balanced gender distribution and varied BMI classifications (Gellish et al., 2007). Health status diversity ranged from healthy individuals to those with multiple comorbidities including diabetes, hypertension, and controlled cardiovascular conditions (Fletcher et al., 2018). Exercise experience varied from sedentary to regular exercisers, enabling assessment of models’ adaptability to different baseline fitness levels. Each profile contained standardized data fields critical for exercise prescription decisions (Lobelo et al., 2018), including anthropometrics, chronic disease information, medication regimens with exercise implications, and specific exercise capacity limitations. Exercise-related factors encompassed current activity patterns, preferences, and previous adverse reactions to physical activity (Campbell et al., 2019). This comprehensive approach reflected best practices in clinical exercise prescription as outlined by Riebe et al (Magal and Riebe, 2016). Profiles underwent a three-stage validation process to ensure clinical realism. Initial drafts were developed using epidemiological data for population representativeness (White and Carlin, 2010). Two sports medicine students then independently reviewed each profile for clinical validity. Finally, a sports medicine expert conducted a comprehensive assessment (Zamanzadeh et al., 2015), rating profiles on authenticity and clinical relevance. Profiles not meeting validation thresholds were refined to ensure realistic clinical scenarios. The complete characteristics of the synthetic patient profiles are presented in **Table 1**. To illustrate the comprehensive nature of patient profiles used in this study, a detailed example of patient demographics and corresponding AI-generated exercise prescription is provided in **Supplementary Table 5**.

**Table 1:** Characteristics of synthetic patient profiles (N = 30).

Characteristic	Value
Demographics	
Age, years (mean ± SD)	52.3 ± 15.8
Age range	20-75
Gender, n (%)	
Female	16 (53.3%)
Male	14 (46.7%)
Anthropometric data	
Body mass index (BMI), kg/m² (mean ± SD)	26.4 ± 4.2
BMI classification, n (%)	
Normal weight (18.5-24.9)	12 (40.0%)
Overweight (25.0-29.9)	11 (36.7%)
Obese (≥30.0)	7 (23.3%)
Health status	
Chronic conditions, n (%)	
Type 2 diabetes	8 (26.7%)
Hypertension	10 (33.3%)
Cardiovascular disease	6 (20.0%)
Musculoskeletal disorders	9 (30.0%)
Multiple comorbidities	5 (16.7%)
Healthy individuals	9 (30.0%)
Exercise background	
Current activity Level, n (%)	
Sedentary	11 (36.7%)
Irregularly active	13 (43.3%)
Regular exercisers	6 (20.0%)
Exercise capacity limitations, n (%)	14 (46.7%)
Previous exercise-related adverse events, n (%)	7 (23.3%)
Medications with exercise implications, n (%)	12 (40.0%)

### 2.4 Model input design

Four LLMs were evaluated using their official APIs in April 2025 (specifically April 15-20, 2025): GPT-4o (OpenAI), Claude 3.7 (Anthropic), DeepSeek R1 (DeepSeek), and Grok-3 (xAI). To ensure unbiased evaluation, each model was accessed through a newly created account without prior conversation history. Generation parameters were standardized across all models: temperature=0.7, max_tokens=2000, top_p=0.95, following established practices for clinical LLM applications (Hu et al., 2024; Windisch et al., 2024). Each prescription was generated in a single API call without iterative refinement to simulate real-world clinical use. The prompt structure consisted of a system message defining the AI as a certified exercise physiologist adhering to ACSM guidelines, and a user message containing patient information with explicit FITT-VP requirements. The development of standardized prompt templates for this study followed a systematic approach to ensure consistency across all model evaluations while maximizing the quality of generated exercise prescriptions. The prompt engineering process was guided by established principles for eliciting structured outputs from large language models, with particular attention to healthcare applications (Silva et al., 2022). As noted by Liu et al. (2023), effective prompt design for clinical applications requires clear task specification, domain-specific contextual information, and explicit output format instructions.

The prompt template development process involved three sequential stages. First, a comprehensive review of exercise prescription literature was conducted to identify the essential components that should be included in FITT-VP-based prescriptions. Second, preliminary prompt variations were tested with each model to assess response patterns and identify potential areas of confusion or ambiguity. Finally, the prompt structure was refined through an iterative process until it consistently elicited comprehensive exercise prescriptions across all models under evaluation (Hu et al., 2024; Liévin et al., 2024).

### 2.5 Expert rating standards

Given the large sample size, we developed a standardized evaluation form based on guidelines from the American College of Sports Medicine (ACSM), American Heart Association (AHA), and World Health Organization (WHO). This comprehensive scoring instrument provided specific assessment criteria for each FITT-VP dimension with detailed benchmarks for scoring. To maintain evaluation quality and prevent assessor fatigue, we implemented a batch assessment approach where experts evaluated subsets of prescriptions in separate sessions (DiPietro et al., 2020). Three certified exercise specialists independently rated each prescription using a 0–10 numerical rating scale, with explicit scoring guidelines for frequency appropriateness, intensity prescription accuracy, time duration appropriateness, exercise type clarity, volume personalization, and progression adaptation. To minimize evaluation bias, expert raters were blinded to model identities during the assessment process, with all prescriptions presented in randomized order using coded identifiers that concealed their AI source. The evaluation form included specific reference standards for each score level to enhance objectivity and consistency. Prior to formal assessment, raters completed calibration sessions with sample prescriptions to ensure scoring alignment with established exercise prescription guidelines (van Hulst et al., 2009). All dimensional assessments evaluated alignment with clinical guideline recommendations (ACSM, AHA, WHO) rather than direct clinical safety outcomes, with lower scores indicating deviation from evidence-based standards rather than validated safety risks. Inter-rater reliability was assessed using intraclass correlation coefficient (ICC), which demonstrated excellent agreement among the three raters (ICC = 0.94, 95% CI: 0.91-0.96), confirming the consistency and reliability of the evaluation process. The comprehensive evaluation framework and detailed scoring guidelines are presented in **Supplementary Table 7**, while the specific evaluation form used by raters is presented in **Supplementary Table 8**.

### 2.6 Statistical analysis

Statistical analysis was conducted using R version 4.3.0 (R Foundation for Statistical Computing, Vienna, Austria) with significance set at α = 0.05. All data were complete with no missing values across the 120 evaluations (30 patient profiles × 4 models). Prior to primary analysis, data normality was assessed using the Shapiro-Wilk test for FITT-VP total scores within each model group, and homogeneity of variance was evaluated using Levene’s test. The primary analysis employed a one-way repeated measures ANOVA to compare mean FITT-VP total scores across the four language models (Claude 3.7, Grok-3, GPT-4o, and DeepSeek R1), with each of the 30 patient profiles serving as its own control across model evaluations. The dependent variable was the total FITT-VP score (range: 0-60), calculated as the sum of six dimensional scores (each rated 0-10). Following significant omnibus ANOVA results, pairwise comparisons were conducted using paired t-tests with Bonferroni correction to control for Type I error inflation, resulting in a corrected significance level of α = 0.0083 (0.05/6 comparisons). Effect sizes were calculated using partial eta-squared (η²) for the omnibus ANOVA and Cohen’s d for pairwise comparisons, with interpretations following established conventions: small (d = 0.2), medium (d = 0.5), and large (d = 0.8) effects. Ninety-five percent confidence intervals (95% CI) were calculated for all pairwise mean differences using the t-distribution with degrees of freedom equal to n-1 (df = 29). Secondary analyses examined performance across individual FITT-VP dimensions using the same statistical framework, with descriptive statistics (mean ± standard deviation) calculated for each model’s performance on the six dimensions. The repeated measures design effectively controlled for patient-specific factors while isolating model performance differences, enhancing the internal validity of comparisons.

## 3 Results

### 3.1 Model overall performance comparison

Inter-rater reliability among the three expert evaluators was excellent (ICC = 0.94, 95% CI: 0.91-0.96) (**Supplementary Table 1**). Assumption testing confirmed data normality and sphericity (detailed results in **Supplementary Tables 2, 6**), supporting the use of parametric repeated measures ANOVA. **Table 2** presents the statistical comparison results across the four language models, while **Table 3** details the *post-hoc* pairwise comparisons with effect sizes.

**Table 2:** Statistical comparison of four language models on FITT-VP task.

Statistical analysis	Statistic	df	p-value	Effect size	95% CI
One-way ANOVA	F = 250.58	3, 87	< 0.001	η² = 0.896	–

**Table 3:** *Post-hoc* analysis results (bonferroni correction).

Comparison	Mean Diff.	SE	t-value	df	p-value	Cohen’s d	95% CI
Claude 3.7 vs Grok-3	3.14	0.45	6.97	29	< 0.001***	1.27	[2.22, 4.07]
Claude 3.7 vs GPT-4o	6.35	0.41	15.48	29	< 0.001***	2.83	[5.51, 7.19]
Claude 3.7 vs DeepSeek R1	9.97	0.32	15.74	29	< 0.001***	5.01	[9.30, 10.64]
Grok-3 vs GPT-4o	3.21	0.35	9.23	29	< 0.001***	1.69	[2.50, 3.92]
Grok-3 vs DeepSeek R1	6.84	0.36	19.17	29	< 0.001***	3.50	[6.11, 7.57]
GPT-4o vs DeepSeek R1	3.63	0.36	10.21	29	< 0.001***	1.86	[2.91, 4.36]

***p < 0.001 after Bonferroni correction for multiple comparisons (α = 0.0083); Effect size criteria: d = 0.2 (small), d = 0.5 (medium), d = 0.8 (large).

#### 3.1.1 Overall score comparison across FITT-VP dimensions

Based on the 0–10 rating scale (0=lowest, 10=highest) for each dimension, Claude 3.7 demonstrated superior performance across all six FITT-VP dimensions, achieving the highest mean scores in each category (Frequency: 8.52 ± 0.67, Intensity: 8.12 ± 0.72, Time: 8.69 ± 0.65, Type: 8.34 ± 0.89, Volume: 8.10 ± 0.71, Progression: 8.46 ± 0.96) and the highest total mean score (50.23 ± 1.75). This was followed by Grok-3 (47.42 ± 1.50), GPT-4o (44.02 ± 1.68), and DeepSeek R1 (40.30 ± 1.46).

**Figure 1:**
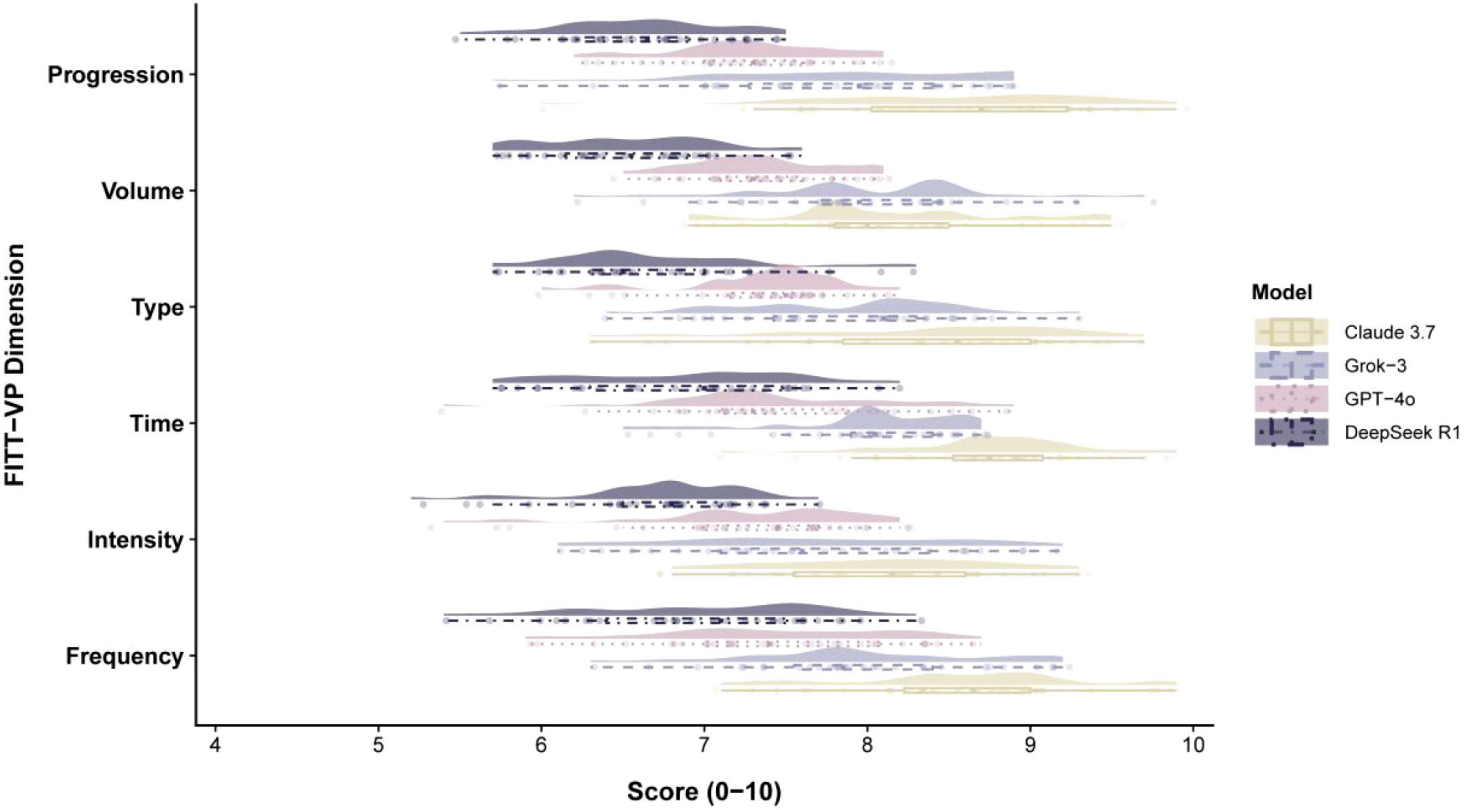
Distribution of FITT-VP scores by model and dimension. Raincloud plots showing distribution of scores (0-10) across FITT-VP dimensions for all evaluated models. Each plot displays individual data points, probability density distributions, and box plots (median with interquartile ranges). Claude 3.7 (beige) demonstrates consistently higher scores across all dimensions, particularly in Time and Progression, followed by Grok-3 (purple-blue), while GPT-4o (rose pink) shows moderate performance and DeepSeek R1(deep purple) consistently scores lower across all dimensions.

One-way ANOVA revealed significant differences among the four models (F(3,87)=250.58, p<0.001, η²=0.896), indicating that model type explained 89.6% of the variance in FITT-VP scores. *Post-hoc* analysis with Bonferroni correction showed significant differences between Claude 3.7 and both GPT-4o (t(29)=15.48, p<0.001, d=2.83) and DeepSeek R1 (t(29)=15.74, p<0.001, d=5.01), with large effect sizes indicating substantial practical differences. The difference between Claude 3.7 and Grok-3 was statistically significant but with a more moderate effect size (t(29)=6.97, p<0.001, d=1.27), suggesting closer performance between these two models. The distribution patterns across all FITT-VP dimensions are illustrated in **Figure 1**.

### 3.2 Model performance characteristics and patterns

#### 3.2.1 Dimensional performance analysis

Detailed examination of individual FITT-VP dimensions revealed specific performance patterns for each model. In the Frequency dimension, Claude 3.7 exhibited the most accurate prescriptions, followed by Grok-3, GPT-4o, and DeepSeek R1. For Intensity, Claude 3.7 consistently provided appropriate prescriptions with proper heart rate ranges and RPE scales while other models showed varying limitations. The Time dimension demonstrated the greatest separation between models, with Claude 3.7 providing detailed session duration recommendations including proper warm-up and cool-down periods. For exercise Type, both Claude 3.7 and Grok-3 demonstrated appropriate modality recommendations while GPT-4o showed moderate performance and DeepSeek R1 frequently recommended generic exercise types. Volume recommendations showed Claude 3.7 maintaining high performance with Grok-3 showing competitive results. For Progression planning, Claude 3.7 outperformed other models with detailed advancement strategies, followed by Grok-3, while GPT-4o and DeepSeek R1 demonstrated inadequate long-term progression planning.

#### 3.2.2 Model-specific performance characteristics

Analysis of performance across FITT-VP dimensions revealed distinct patterns for each model. Claude 3.7 demonstrated consistent strength across all dimensions, with particular excellence in Time (mean = 8.69) and Progression (mean = 8.46), suggesting superior capability in planning session durations and developing advancement strategies. Its lowest performance, though still strong, was in Intensity dimension (mean = 8.12). Grok-3 exhibited notable strength in Volume prescriptions (mean = 7.94), nearly matching Claude 3.7 (effect size d = 0.23), but showed weakness in Intensity recommendations (mean = 7.68) where specificity in heart rate zones was often lacking.

**Figure 2:**
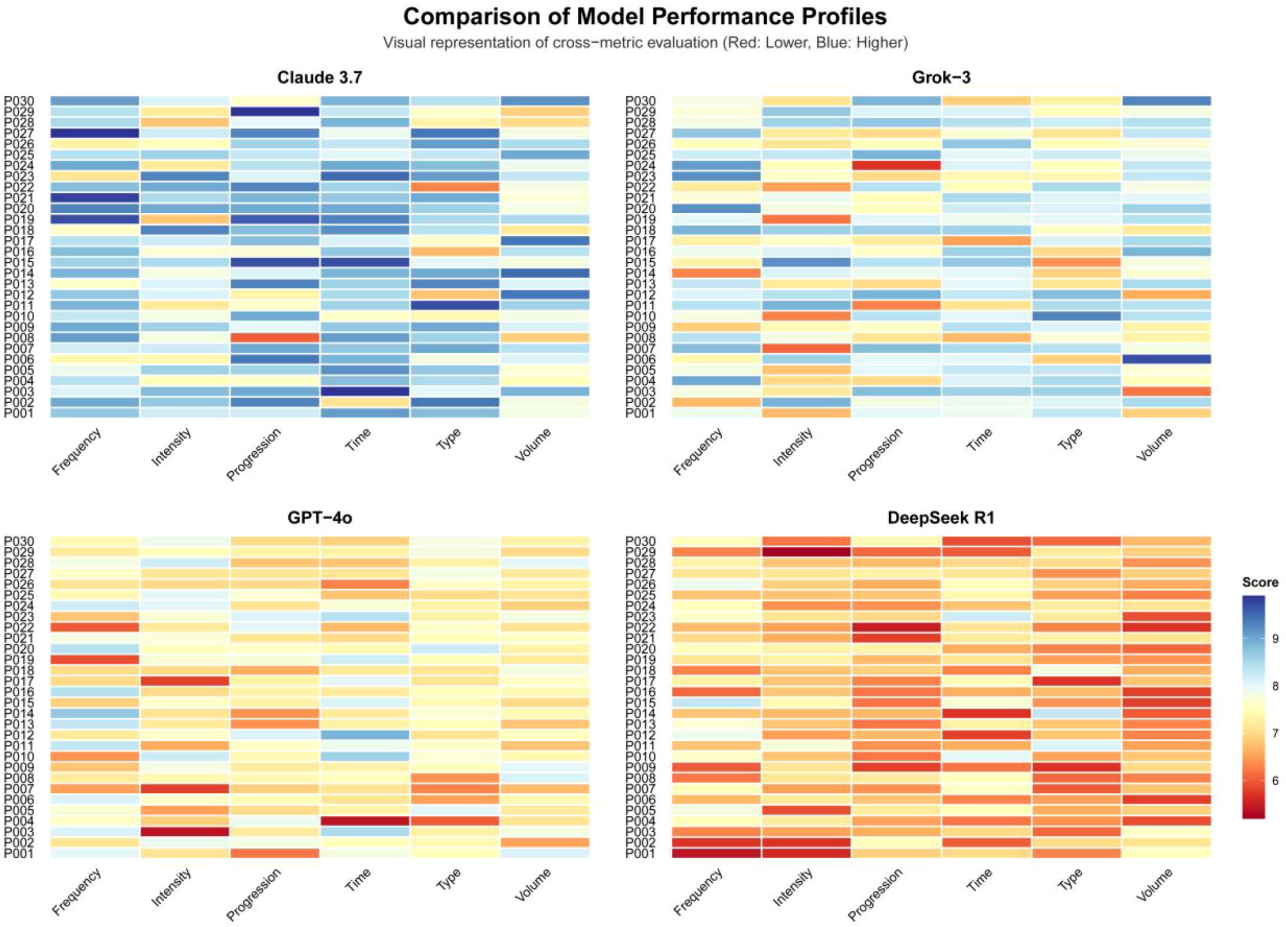
Model performance heatmap. Performance scores (0–10 scale) for four models across 30 patient profiles and six FITT-VP dimensions. Color scale represents performance levels from dark blue (excellent, 9.0-10.0) to red (poor, 5.0-5.9). Fre, Frequency; Int, Intensity; Tim, Time; Typ, Type; Vol, Volume; Pro, Progression.

GPT-4o displayed moderate performance across dimensions, with relative strength in Type (mean = 7.36) but significant weakness in Time dimension (mean = 7.41) with greater variability (SD = 1.14) and several outliers below 6.0, indicating inconsistent ability to structure appropriate session durations. DeepSeek R1 consistently underperformed across all dimensions, with scores clustering in the 6.0-7.0 range, and particular weakness in Intensity (mean = 6.59) and Type (mean = 6.65), suggesting fundamental limitations in its training data or parameter optimization for exercise prescription tasks. See **Figure 2** for model performance heatmap.

## 4 Discussion

The observed performance disparities among the four large language models reveal intriguing patterns across the FITT-VP dimensions. Claude 3.7’s superior performance may suggest more extensive training on healthcare literature and clinical guidelines, or could potentially reflect optimization for temporal and developmental reasoning tasks critical to exercise prescription (Kujala, 2009; Savage et al., 2025). Conversely, DeepSeek R1’s underperformance, especially in Intensity and Type dimensions, may indicate limited exposure to exercise science literature or reflect differences in fine-tuning approaches on exercise prescription tasks (Savage et al., 2025). Beyond training data differences, architectural variations may contribute to performance disparities, as suggested by Touvron et al. (2023). who demonstrated that parameter scale alone does not determine clinical reasoning capabilities; rather, training data quality and domain-specific optimization play crucial roles. The bimodal distribution observed in Grok-3’s performance across patient profiles indicates potential training data imbalances, with stronger performance on common clinical presentations but weaker on complex cases (Agrawal et al., 2019).

The dimensional analysis provides deeper insights into model capabilities. Claude 3.7’s excellence in the Time dimension (mean = 8.69) reflects sophisticated understanding of exercise duration principles, as accurate exercise duration prescription requires complex integration of physiological knowledge and patient-specific factors. The small performance gap in the Volume dimension between Claude 3.7 and Grok-3 suggests that quantitative reasoning about sets, repetitions, and total exercise load appears well-represented in both models’ training, paralleling research by Bansal et al. (Qin et al., 2023) indicating that structured quantitative reasoning may transfer more effectively across model architectures than qualitative clinical reasoning.

These findings have significant implications for AI implementation in exercise medicine, suggesting potential for these systems to serve as clinical decision support tools while underscoring the need for rigorous evaluation frameworks before deployment (Nundy et al., 2019). The ability of AI systems to generate individualized exercise prescriptions presents several advantages over traditional approaches, including rapid processing of multiple patient variables simultaneously and enhanced accessibility to exercise expertise in resource-limited settings (Safi et al., 2018; Detopoulou et al., 2023). Leading models like Claude 3.7 demonstrate robust generalization capabilities across diverse patient profiles, with potential for dynamic prescription adjustment aligning with precision medicine approaches (Roberts et al., 2014). However, significant limitations persist, including inconsistencies in critical areas like intensity prescription, which Roberts et al (Roberts et al., 2014; Rudin, 2019). identified as crucial for optimal clinical outcomes. The decline in performance with complex patient profiles and the “black box” nature of LLM reasoning present challenges for clinical accountability (Gao et al., 2023).

The identified strengths and limitations suggest optimal implementation would involve collaborative frameworks where AI systems augment rather than replace human expertise, leveraging AI strengths while preserving human oversight for safety and clinical judgment (Young et al., 2021). Neri et al. (2019) proposed a “human-in-the-loop” paradigm whereby AI-generated prescriptions serve as evidence-informed starting points that clinicians modify based on patient-specific factors not captured in structured data. Theoretically, this research extends FITT-VP principles into the digital health domain, demonstrating that established exercise science frameworks can effectively structure and evaluate AI-generated content (Shortliffe and Sepúlveda, 2018). The successful application of this framework provides a bridge between traditional exercise science and emerging AI healthcare applications, contributing to broader discussions about knowledge representation in LLMs (Obermeyer and Emanuel, 2016). Practically, our findings provide clear direction for AI-assisted exercise prescription system development, establishing benchmark standards for future systems (Miner et al., 2020), while methodologically introducing a novel approach to evaluating medical AI systems through domain-specific dimensional assessment frameworks that provide granular insights into specific capabilities beyond simple accuracy metrics (De Fauw et al., 2018; Ghassemi et al., 2021).

Several limitations should be acknowledged. First, this study used synthetic patient profiles rather than real clinical data, which may not fully capture the complexity of actual clinical scenarios. Second, the evaluation was conducted by human experts, introducing potential subjectivity despite standardized scoring criteria; moreover, all three expert raters were based in China, which may introduce geographical bias in the interpretation of exercise prescription standards, as regional practice patterns and cultural factors may have influenced evaluation criteria. Third, each prescription was generated through a single API call, and output variability across repeated runs for identical inputs was not empirically examined; given the inherently non-deterministic nature of LLM outputs, this has important implications for clinical reproducibility and professional accountability. Fourth, our evaluation of static documents may not fully capture AI performance in dynamic clinical scenarios, in which real-world exercise prescription typically involves iterative patient feedback, clarification of ambiguous information, and longitudinal modifications based on patient response. Finally, the study focused on prescription quality rather than patient outcomes or adherence rates. Building on these limitations, several concrete directions merit future investigation. First, to address geographical bias, cross-regional validation should incorporate raters from multiple countries with diverse training backgrounds. Second, to strengthen clinical reproducibility, output variability should be systematically quantified through multi-run protocols (≥10 generations per profile per model), with intra-model agreement reported via ICC and score variance. Building on this, dynamic AI performance should be examined through simulated patient–AI dialogue studies evaluating multi-turn adaptation to iterative feedback, thereby extending evaluation from static outputs to interactive clinical reasoning. Finally, downstream evaluation should move beyond prescription quality toward patient-centered endpoints such as adherence, clinical outcomes, and long-term safety, which together represent the ultimate test of clinical utility.

## 5 Conclusion

This study provides a comprehensive assessment of four large language models (Claude 3.7, Grok-3, GPT-4o, and DeepSeek R1) in generating exercise prescriptions using the established FITT-VP framework. Our evaluation across 30 diverse patient profiles revealed significant performance differences between models, with Claude 3.7 showing the strongest performance among evaluated models across all dimensions, particularly in Time and Progression planning. Statistical analysis confirmed significant differences between Claude 3.7 and both GPT-4o and DeepSeek R1, with large effect sizes indicating substantial practical differences. The performance patterns identified specific strengths and weaknesses across models: Claude 3.7 demonstrated strong performance in generating structured prescriptions addressing all FITT-VP dimensions across diverse synthetic patient profiles; Grok-3 showed competitive performance in Volume prescription but struggled with complex comorbidities; GPT-4o demonstrated moderate capabilities but significant inconsistency in Time dimension; and DeepSeek R1 consistently underperformed across all dimensions. These findings establish important comparative performance benchmarks among LLM architectures for drafting initial exercise prescriptions. While these results demonstrate feasibility and suggest promise for assisting in future clinical decision support, substantial further validation is required, including comparison with human-generated prescriptions, assessment of output variability, and evaluation in dynamic clinical settings. Critical human oversight remains essential for all clinical applications. Validation in clinical settings with human–AI interaction and patient follow-up is required before implementation. The dimensional analysis framework introduced here provides a valuable methodology for evaluating AI systems in healthcare beyond simple accuracy metrics.

## Data Availability

The raw data supporting the conclusions of this article will be made available by the authors, without undue reservation.
