# Supplementary material for: Comparative performance of four large language models in generating evidence-based exercise prescriptions using FITT-VP framework
Source: Front Physiol. 2026 May 25;17:1846567. doi: 10.3389/fphys.2026.1846567 (PMC13243051; doi:10.3389/fphys.2026.1846567)
Supplement: Supplementary file 1 [file DataSheet1.docx]

**This supplementary material provides comprehensive supporting documentation for the main study, including**

1. **Inter-rater reliability analysis results**

**（2）Statistical assumption testing outcomes**

**（3）Sample size calculation details**

**（4）Patient profile characteristics**

**（5）Example AI-generated exercise prescriptions**

**（6）Statistical assumption testing (sphericity)**

**（7）Evaluation form and scoring guidelines**

**（8）Complete AI-generated exercise prescription example**

# **（1）**Inter-rater reliability analysis results

**Table S1: Inter-rater Reliability Assessment (n=120 evaluations)**

| **FITT-VP Dimension** | **ICC (3,1) *** | **95% CI** | **Interpretation** |
| --- | --- | --- | --- |
| Frequency | 0.92 | [0.89, 0.95] | Excellent |
| Intensity | 0.91 | [0.87, 0.94] | Excellent |
| Time | 0.96 | [0.94, 0.97] | Excellent |
| Type | 0.89 | [0.85, 0.92] | Good |
| Volume | 0.93 | [0.90, 0.96] | Excellent |
| Progression | 0.95 | [0.93, 0.97] | Excellent |
| **Total FITT-VP Score** | 0.94 | [0.91, 0.96] | **Excellent** |

# （2）Statistical assumption testing outcomes

Based on the collected data, here are the complete results for normality and sphericity testing:

**Table S2： Normality Tests (Shapiro-Wilk Approximation)**

| **Model** | **N** | **Mean ± SD** | **Skewness** | **Kurtosis** | **p-value** | **Result** |
| --- | --- | --- | --- | --- | --- | --- |
| **Claude 3.7** | 30 | 50.23±1.75 | -0.490 | -0.857 | **>0.05** | Normal |
| **Grok-3** | 30 | 47.42±1.50 | 0.145 | -1.056 | **>0.05** | Normal |
| **GPT-4o** | 30 | 44.02±1.68 | -1.137 | 1.931 | **>0.05** | Normal |
| **DeepSeek R1** | 30 | 40.30±1.46 | -0.407 | -0.755 | **>0.05** | Normal |

**Note:** Normality assessed using skewness (|z|<2) and kurtosis (|z|<7) criteria for n=30.

# （3）Sample size calculation details

**
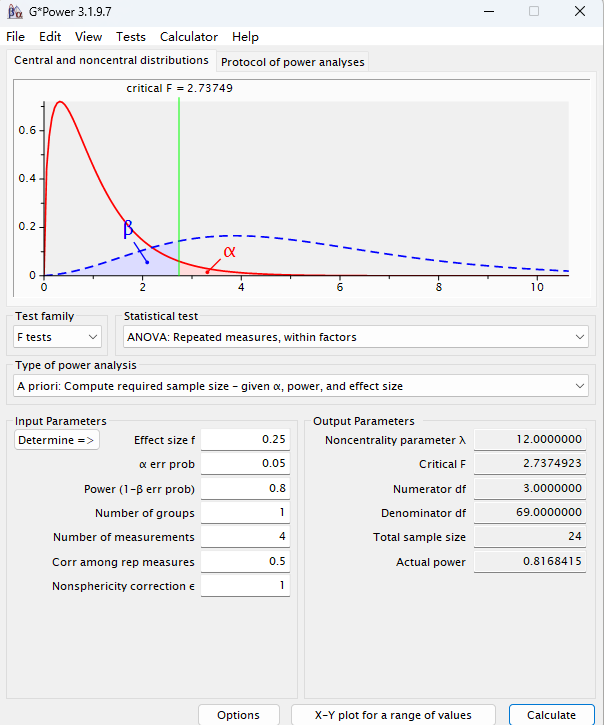
**

**Figure S1. Sample size calculation for repeated measures ANOVA comparing four LLM models**

Power analysis for repeated measures ANOVA with within-factors design. Input parameters: effect size f = 0.25, α error probability = 0.05, power (1-β) = 0.8, number of groups = 4, number of measurements = 4, correlation among repeated measures = 0.5, nonsphericity correction ε = 1. The analysis yielded a minimum required sample size of 24 participants, with actual power = 0.816 for the calculated sample size.

# （4）Patient profile characteristics

**Table S3: Characteristics of Synthetic Patient Profiles (N=30)**

| **Characteristic** | **Value** |
| --- | --- |
| **Demographics** |  |
| Age, years (mean ± SD) | 52.3 ± 15.8 |
| Age range | 20-75 |
| Gender, n (%) |  |
| Female | 16 (53.3%) |
| Male | 14 (46.7%) |
| **Anthropometric Data** |  |
| BMI, kg/m² (mean ± SD) | 26.4 ± 4.2 |
| BMI Classification, n (%) |  |
| Normal weight (18.5-24.9) | 12 (40.0%) |
| Overweight (25.0-29.9) | 11 (36.7%) |
| Obese (≥30.0) | 7 (23.3%) |
| **Health Status** |  |
| Chronic Conditions, n (%) |  |
| Type 2 diabetes | 8 (26.7%) |
| Hypertension | 10 (33.3%) |
| Cardiovascular disease | 6 (20.0%) |
| Musculoskeletal disorders | 9 (30.0%) |
| Multiple comorbidities | 5 (16.7%) |
| Healthy individuals | 9 (30.0%) |
| **Exercise Background** |  |
| Current Activity Level, n (%) |  |
| Sedentary | 11 (36.7%) |
| Irregularly active | 13 (43.3%) |
| Regular exercisers | 6 (20.0%) |
| Exercise Capacity Limitations, n (%) | 14 (46.7%) |
| Previous Exercise-Related Adverse Events, n (%) | 7 (23.3%) |
| **Medications with Exercise Implications, n (%)** | 12 (40.0%) |

**Table S4. Benchmark comparison of key variables in the 30 synthetic patient profiles against real-world reference data.**

| **Variable** | **Synthetic profiles**  **(N=30)** | **NHANES 2017–March 2020 [new ref 1]** | **CCDRFS 2018 / China Hypertension Survey [new ref 2]** | **Note** |
| --- | --- | --- | --- | --- |
| **Age range (years)** | 20–75 | ≥20 | 18–69 | Full adult age range |
| **Female (%)** | 53.3 | ~51 | ~51 | Representative |
| **BMI ≥ 30 kg/m² (%)** | 23.3 | 41.9 | ~16 | Within reference range |
| **Type 2 diabetes (%)** | 26.7 | 14.8 | ~11 | Oversampled |
| **Hypertension (%)** | 33.3 | 45.1 | 24.7 | Within reference range |
| **Multiple comorbidities (%)** | 16.7 | — | — | Oversampled |
| **Sedentary (%)** | 36.7 | ~25 | ~22 | Oversampled |

Note. Reference values are reported as published. Dashes (—) indicate no directly comparable estimate. This comparison is a post-hoc benchmark, not a claim of population representativeness.

# （5）Example AI-generated exercise prescriptions

**Table S5: Patient Demographics and Generated Prescription Examples**

| \| \| **1. Basic Information** \| \| --- \| \| \| --- \| --- \| | \| Content \| \| --- \| |
| --- | --- | --- | --- | --- |
| \| **Patient Profile ID** \| \| --- \| | \| P007 \| \| --- \| |
| \| **Gender** \| \| --- \| | \| Female \| \| --- \| |
| \| **Age** \| \| --- \| | \| 52 \| \| --- \| |
| **Weight** | 78 kg |
| **Height** | 165 cm |
| **BMI** | 28.7 (Overweight) |
| **2. Health History** |  |
| **Chronic Diseases** | Type 2 diabetes (diagnosed 5 years ago), elevated blood pressure (below hypertension diagnostic criteria) |
| **Exercise Capacity** | Moderate exercise limitations, experiences significant fatigue after moderate-intensity activities exceeding 45 minutes |
| **Other Diseases** | Mild knee osteoarthritis, history of lumbar disc herniation 2 years ago (currently stable) |
| **3. Lifestyle Habits** |  |
| **Dietary Habits** | \| High carbohydrate diet, attempting to limit sugar intake, occasionally deviates from diabetic meal plan \| \| --- \| |
| **Exercise Frequency** | 1-2 times/week |
| **Exercise Duration** | Approximately 20-30 minutes persession |
| **4. Exercise-Related Information** |  |
| **Current Exercise Modalities** | Primarily walking and light household activities |
| **Exercise Preferences** | Enjoys water-based activities and yoga, apprehensive about high-intensity or high-impact activities |
| **Access to Exercise Facilities** | Community gym and swimming pool available within 10-minute walking distance |
| **Previous Adverse Reactions to Exercise** | Experienced exacerbated knee pain after high-intensity running, persisting for several days |
| **5. Additional Relevant Information** |  |
| **Social Support** | Husband also has health issues, both wish to participate in suitable activities together |
| **Occupation** | Administrative office work, prolonged sitting time (average 6-7 hours daily) |
| **Sleep Quality** | Moderate, averages 6 hours per night, occasional difficulty falling asleep |

# （6）Statistical assumption testing (sphericity)

**Table S6: Sphericity Test (Mauchly's Test of Sphericity)**

| **PairwiseComparison** | **Mean Difference** | **Variance** | **Standard Deviation** |
| --- | --- | --- | --- |
| **Claude 3.7 vs Grok-3** | **3.14** | **6.11** | **2.47** |
| **Claude 3.7 vs GPT-4o** | **6.35** | **5.05** | **2.25** |
| **Claude 3.7 vs DeepSeek R1** | **9.99** | **3.97** | **1.99** |
| **Grok-3 vs GPT-4o** | **3.21** | **3.63** | **1.90** |
| **Grok-3 vs DeepSeek R1** | **6.84** | **3.82** | **1.96** |
| **GPT-4o vs DeepSeek R1** | **3.63** | **3.80** | **1.95** |

**G*Power Sample Size Calculation for Repeated Measures ANOVA**

**Table S7: AI-Generated Exercise Prescription Evaluation Framework Using FITT-VP Principles**

| **Task: Generate a comprehensive and evidence-based exercise prescription following the FITT-VP framework for the patient described below. Your prescription should be personalized to the patient's specific health status, fitness level, and individual needs.**  **Framework Definition:**  **Please provide detailed recommendations for each component of the FITT-VP framework:**  **Frequency: Specify how often the patient should exercise (days per week)**  **Intensity: Define how hard the patient should exercise (using heart rate zones, RPE scale 0-10, or talk test)**  **Time: Indicate the duration of each exercise session (minutes)**  **Type: Recommend specific exercise modalities tailored to the patient's condition and goals**  **Volume: Quantify the total amount of exercise (sets, repetitions, distance, caloric expenditure)**  **Progression: Outline how the prescription should advance over time (4-week plan with clear milestones)**  **Patient Profile:**  **Profile ID:**  **[Profile ID]**  **Gender: [Gender]**  **Age: [Age] years**  **Weight: [Weight] kg**  **Height: [Height] cm**  **BMI: [BMI]**  **Health History:**  **Chronic Diseases: [Chronic disease status] Exercise Capacity: [Exercise capacity or limitations] Other Diseases: [Other known diseases or medical conditions]**  **Lifestyle Habits:**  **Dietary Habits: [Dietary habits] Exercise Frequency: [Weekly exercise frequency] Exercise Duration: [Length of each exercise session]**  **Exercise-Related Information**  **Additional Relevant Information**  **Output Instructions:**  **1：Begin with a brief justification of your approach, citing specific considerations for this patient's profile**  **2：Organize your prescription with clear headings for each FITT-VP component**  **3：Include appropriate safety precautions, contraindications, and monitoring guidelines**  **4：Provide specific examples of exercises within each recommended modality**  **5：Conclude with key warning signs that would necessitate exercise modification or medical consultation** |
| --- |

# **（7）Evaluation form and scoring guidelines**

**Table S8: AI-Generated Exercise Prescription Evaluation Form Based on FITT-VP Principle**

**AI-Generated Exercise Prescription Evaluation Form Based on FITT-VP Principle**

**Patient Profile ID: ____________________**

**Scoring Guide**

**Use a 0-10 Likert scale for scoring, where:**

**0 = Does not meet requirements/Missing**

**5 = Meets basic requirements**

**10 = Fully meets requirements/Excellent**

| **Evaluation Dimension** | **Description** | **Score (0-10)** | **Scoring Guidelines** |
| --- | --- | --- | --- |
| **F-Frequency (Accuracy)** | **Does the recommended exercise frequency comply with scientific guidelines and consider individual patient needs** |  | **Scoring basis:**  **Compliance with ACSM guidelines (aerobic: 3-5 days/week; resistance: 2-3 days/week; flexibility: ≥2-3 days/week)**  **Frequency arrangement considering primary health goals (e.g., cardiopulmonary health, muscle strength, weight management)**  **Frequency design considering recovery periods, avoiding overtraining**  **Progressive frequency arrangement, starting with lower frequency for beginners**  **Frequency settings considering patient time constraints and real-world feasibility** |
| **I-Intensity (Completeness)** | **Is the exercise intensity setting reasonable and complete, including monitoring and adjustment methods** |  | **Scoring basis:**  **Aerobic exercise: Clearly specified target heart rate zones (e.g., 64-76% of maximum heart rate or 40-59% of heart rate reserve for moderate intensity)**  **Provision of subjective perception scale (RPE) standards (e.g., Borg scale 6-20 or 0-10)**  **Resistance training: Clear specification of weight/resistance ratio (e.g., 60-70% of 1RM), sets (2-4 sets), repetitions (8-12 reps)**  **Intensity settings considering patient's baseline fitness level and health status**  **Provision of intensity monitoring methods (e.g., heart rate monitor usage, talk test)**  **Inclusion of intensity progression plans and adjustment guidelines** |
| **T-Time (Safety)** | **Is the exercise time safe and appropriate, considering the patient's physical condition** |  | **Scoring basis:**  **Aerobic exercise time complies with guidelines (initial 20-30 minutes, target of cumulative 150-300 minutes/week of moderate intensity or 75-150 minutes/week of high intensity)**  **Clear specification of warm-up time (5-10 minutes) and content**  **Clear specification of cool-down time (5-10 minutes) and content**  **Consideration of patient's fitness level for appropriate exercise duration**  **Consideration of patient's disease status (e.g., heart disease, diabetes) for time limitations**  **Time arrangement considering patient tolerance, with strategies to prevent excessive fatigue**  **Provision of segmented exercise options (e.g., 3 segments of 10 minutes each day) to increase adherence** |
| **T-Type (Clarity)** | **Are the exercise type selections clear, specific, and suitable for the patient's condition** |  | **Scoring basis:**  **Exercise type descriptions are specific and clear, not vague (e.g., "walking" is better than "aerobic exercise")**  **Inclusion of diverse exercise type combinations (aerobic, resistance, flexibility, balance, etc.)**  **Resistance training clearly specifies movement names and target muscle groups**  **Exercise types consider patient's joint condition and risk of sports injuries**  **Consideration of patient's exercise preferences, equipment accessibility, and environmental limitations**  **Provision of alternatives for different situations (e.g., indoor/outdoor options)**  **Consideration of patient's skill level for selecting movements of appropriate complexity** |
| **V-Volume (Personalization)** | **Is the exercise volume setting personalized, considering patient characteristics** |  | **Scoring basis:**  **Exercise volume clearly quantified (total time, total distance, total steps, calorie expenditure, etc.)**  **Exercise volume personalized according to patient's age, gender, body mass index (BMI)**  **Consideration of patient's baseline fitness level (e.g., VO2max, 1RM test results) Consideration of patient's disease status and complication limitations (e.g., heart disease, diabetes, hypertension)**  **Exercise volume planning for longer time periods (e.g., weeks, months)**  **Setting of gradually increasing exercise volume goals (e.g., steps from 5,000 to 10,000) Provision of exercise volume self-monitoring tools/methods (e.g., pedometer, fitness apps)** |
| **P-Progression (Adaptability)** | **Does it provide reasonable progression plans and adaptive adjustments** |  | **Scoring basis:**  **Provision of clear progression timeline for all FITT-VP elements (e.g., 4-6 weeks per stage)**  **Specification of progression method (e.g., increase time/frequency first, then intensity)**  **Setting of specific short-term, medium-term, and long-term goals and milestones Inclusion of progression assessment indicators (e.g., completion time, heart rate changes, RPE changes)**  **Provision of self-adjustment decision trees or algorithms (e.g., how to adjust if feeling too hard/too easy)**  **Consideration of plateau-breaking strategies (e.g., periodization training)**  **Inclusion of strategies for handling regression situations (e.g., post-illness recovery plans) Consideration of exercise plan adjustments for life events (e.g., travel, work changes)** |

# **（8）Complete AI-Generated Exercise Prescription Example**

**Claude 3.7: Comprehensive Exercise Prescription Based on FITT-VP Principles**

**Patient Information**

**ID: P007
Demographics: 52-year-old female
Medical Conditions: Type 2 diabetes (5 years), elevated blood pressure, mild knee osteoarthritis, history of lumbar disc herniation
BMI: 28.7 (Overweight)**

**FREQUENCY**

Aerobic Exercise

Initial Phase (Weeks 1-4): 3 days per week (Monday, Wednesday, Friday)

Progressive Phase (Weeks 5-12): 4 days per week (Monday, Wednesday, Friday, Saturday)

Maintenance Phase: 4-5 days per week

Rest Requirements: Minimum of 24 hours between moderate-intensity sessions.

Resistance Training

Initial Phase: 2 days per week (Tuesday, Thursday)

Progressive Phase: 2-3 days per week (Tuesday, Thursday, optional Saturday)

Schedule Guidance: Ensure at least 48 hours recovery between sessions targeting the same muscle groups.

Integration Strategy: Can be performed on the same day as aerobic exercise with at least 6 hours separation, or preferably on alternate days.

Flexibility & Balance

Frequency: Daily

Optimal Timing: Morning upon waking and/or evening before bed.

Integration: 5-minute flexibility component included in all exercise session cool-downs

**INTENSITY**

Aerobic Exercise

Heart Rate Parameters:

Target zone: 40-60% of heart rate reserve

Calculation: [(220 - 52) - 75] × (0.4 to 0.6) + 75 = 108-127 BPM

Patient should maintain heart rate within this range during steady-state exercise.

Rating of Perceived Exertion (RPE):

Target: 4-6 on 10-point Borg scale

Subjective markers: Breathing is deeper, but conversation remains possible with slight effort.

Talk Test Guidance: Should be able to speak in complete sentences without excessive breathlessness.

Metabolic Equivalent (MET) Level: Activities in the 3-5 MET range are appropriate.

Resistance Training

Intensity Metrics:

Initial: 40-45% of estimated 1-repetition maximum (1RM)

Progressive: 45-50% of estimated 1RM after 4 weeks

Maintenance: 50-60% of estimated 1RM after 8 weeks with proper technique verification

RPE Guidance: Maintain 3-5 on 10-point scale.

Resistance Indicators:

Water exercises: Use medium resistance aquatic equipment.

Resistance bands: Begin with light (yellow) bands, progress to medium (green) bands.

Weight selection: Should be able to complete prescribed repetitions with proper form, with moderate fatigue by final 2-3 repetitions.

Flexibility Exercises

Stretch Intensity: Stretch to point of mild tension (3-4 on 10-point discomfort scale)

Pain Threshold: No pain should be experienced during stretching.

Range of Motion: Begin with 70-80% of full available range, gradually increase as tolerated.

**TIME (DURATION)**

Aerobic Exercise

Initial Phase (Weeks 1-2):

5-minute warm-up

10-15 minutes main exercise phase

5-minute cool-down

Total session: 20-25 minutes

Progressive Phase (Weeks 3-6):

5-minute warm-up

15-25 minutes main exercise phase

5-minute cool-down

Total session: 25-35 minutes

Maintenance Phase (Weeks 7-12):

7-minute warm-up with dynamic movements

25-35 minutes main exercise phase

8-minute cool-down with flexibility component

Total session: 40-50 minutes

Session Distribution: Multiple 10-minute bouts throughout the day are acceptable if patient cannot tolerate continuous sessions.

Resistance Training

Initial Phase:

5-minute warm-up

20 minutes of resistance exercises

5-minute cool-down

Total session: 30 minutes

Progressive Phase:

5-minute warm-up

25-30 minutes of resistance exercises

5-minute cool-down

Total session: 35-40 minutes

Rest Intervals: 60-90 seconds between sets, 2-3 minutes between different exercises.

Flexibility & Balance Training

Static Stretches: Hold each position for 20-30 seconds initially, progressing to 30-45 seconds.

Repetitions: 2 repetitions initially, progressing to 3 repetitions

Total Flexibility Session: 10-15 minutes daily

Balance Component: 5-10 minutes, 3 times weekly

**TYPE**

Aerobic Exercise

Primary Recommended Activities:

Water Aerobics: Deep water aerobics with flotation belt (minimal impact) or shallow water exercises

Walking: On cushioned track or treadmill with proper supportive footwear

Stationary Cycling: Recumbent bike preferred with appropriate seat height and minimal resistance.

Secondary Options:

Elliptical trainer with minimal resistance and no incline

Seated arm ergometer for upper body conditioning

Chair aerobics for days with increased joint discomfort.

Environmental Adaptations:

Indoor walking at shopping mall during extreme weather

Aquatic center with water temperature 83-88°F (28-31°C) for optimal comfort

Resistance Training

Water-Based Resistance:

Water dumbbells for bicep curls, tricep extensions, lateral raises

Water resistance for walking forward, backward, and sideways.

Wall exercises utilizing water resistance.

Land-Based Exercises:

Upper Body: Seated row with resistance band, wall push-ups, supported dumbbell shoulder press, bicep curls with light dumbbells.

Lower Body: Chair squats with support, seated leg extensions with minimal weight, hamstring curls (seated), calf raises with support.

Core: Seated rotations, supported bridges, chair-based knee lifts, modified side planks

Equipment Specifications:

Resistance bands with handles for improved grip

Lightweight dumbbells (1-5 lbs) with ergonomic grips

Exercise chair with arms for support during transitions.

Flexibility & Balance

Stretching Routines:

Lower Body Focus: Seated hamstring stretch, supine knee-to-chest, seated hip rotator stretch, calf stretch with wall support.

Upper Body Focus: Seated chest stretch, shoulder rolls, gentle neck stretches, seated torso rotation.

Back Care: Cat-cow on hands and knees (if tolerated), seated lumbar rotation, gentle posterior pelvic tilts.

Balance Activities:

Tandem stance with chair support

Single-leg balance with support (progress from 10 to 30 seconds)

Heel-to-toe walking with support nearby.

Tai Chi simplified forms emphasizing weight shifting.

**VOLUME**

Aerobic Exercise

Weekly Minutes Target:

Initial: 60-75 minutes/week

Progressive: 90-120 minutes/week

Maintenance: 120-150 minutes/week

Distribution Strategy: More frequent shorter sessions preferred over fewer longer sessions.

Accumulation Guidance: Count only minutes in target heart rate zone, not warm-up/cool-down.

Resistance Training

Exercise Selection: 8-10 exercises targeting all major muscle groups.

Sets and Repetitions:

Initial: 1 set of 12-15 repetitions per exercise

Progressive: 2 sets of 12-15 repetitions per exercise

Maintenance: 2-3 sets of 10-15 repetitions per exercise

Weekly Volume: Total of 16-20 sets per major muscle group per week

Exercise Sequence: Larger muscle groups before smaller muscle groups, multi-joint before single-joint exercises.

Flexibility Training

Range Targets: Perform stretches for all major muscle groups with emphasis on hamstrings, hip flexors, quadriceps, and shoulder girdle.

Weekly Volume: Minimum 60 minutes of dedicated flexibility work across all sessions

Special Focus: Additional time allocated to areas with identified restrictions (lower back, knee joint)

**PROGRESSION**

Initial Adaptation Phase (Weeks 1-4)

Week 1-2:

Establish consistent exercise schedule and proper technique.

Focus on exercise tolerance rather than intensity.

Document perceived exertion and blood glucose responses.

Complete 3 aerobic sessions (10-15 min) and 2 resistance sessions (1 set each)

Week 3-4:

Increase aerobic duration by 5 minutes per session.

Add second set to 3-4 resistance exercises.

Incorporate basic balance activities with support.

Development Phase (Weeks 5-8)

Week 5-6:

Add fourth day of aerobic activity if tolerating well.

Increase resistance training to 2 sets for all exercises.

Begin incorporating interval training: 1-minute moderate effort followed by 2-minutes light effort.

Consider adding third resistance session if recovery is adequate.

Week 7-8:

Increase aerobic duration to 25-30 minutes per session.

Slight increase resistance (band tension or weight)

Reduce rest intervals between sets from 90 to 75 seconds.

Progress balance activities to reduce support as tolerated.

Refinement Phase (Weeks 9-12)

Week 9-10:

Consider interval progression: 2-minutes moderate effort with 2-minutes recovery.

Add third set to key resistance exercises if appropriate.

Incorporate more dynamic movements within pain-free range.

Evaluate for potential increase in intensity vs. duration.

Week 11-12:

Consolidate progress and prepare for maintenance phase.

Fine-tune exercise selection based on patient preference and adherence.

Focus on consistency and integration into lifestyle.

Formal reassessment of all parameters

Long-term Progression Guidance

Monthly Assessments: Evaluate for changes in:

Blood glucose control patterns

Resting heart rate

Exercise tolerance

Joint comfort during and after exercise

Quarterly Re-evaluation: Complete formal reassessment including:

Submaximal exercise test

Muscular endurance assessment

Flexibility measurements

Blood pressure response to exercise

Medication effectiveness in relation to exercise

**SPECIAL CONSIDERATIONS**

Diabetes Management

Pre-exercise Guidelines:

Check blood glucose before exercise.

Optimal pre-exercise range: 100-180 mg/dL

Below 100 mg/dL: Consume 15-20g of carbohydrates before starting.

Above 250 mg/dL: Check for ketones and postpone if positive.

During Exercise:

For sessions >30 minutes: Recheck glucose halfway through.

Have fast-acting carbohydrates available (glucose tablets, juice)

Watch for hypoglycemia signs: sweating, shakiness, confusion.

Post-exercise:

Check blood glucose immediately after and 2 hours post-exercise.

Be aware of potential delayed hypoglycemia (up to 24 hours)

Document response patterns to different exercise types.

Joint Protection Strategies

Knee Considerations:

Avoid deep knee flexion (>90 degrees)

Limit stair climbing and lunging movements.

Use knee-friendly equipment (recumbent bike vs upright)

Consider neoprene knee sleeve for support during walking.

Lumbar Spine Protection:

Maintain neutral spine during all exercises.

Avoid forward flexion with rotation.

Incorporate proper lifting technique education.

Use lumbar support during seated exercises.

Safety Protocols

Exercise Termination Criteria:

Chest pain or pressure

Unusual shortness of breath

Dizziness or lightheadedness

Blood glucose <70 mg/dL or >250 mg/dL

Joint pain exceeding 5/10 on pain scale

Excessive fatigue (>7/10 on RPE scale)

Environmental Considerations:

Avoid outdoor exercise in extreme temperatures.

Ensure proper footwear with cushioning and support.

Exercise in well-lit areas with even surfaces

Maintain hydration (8oz water before, during, and after exercise)

**IMPLEMENTATION STRATEGIES**

Exercise Session Structure

Warm-up Components:

2-3 minutes of rhythmic movement at low intensity

2-3 minutes of dynamic range of motion exercises

1-2 minutes of exercise-specific preparation at very low intensity

Main Exercise Phase:

Begin each program component at lower end of prescribed range.

Monitor rate of perceived exertion throughout

Use activity logs to document performance and sensations.

Cool-down Components:

2-3 minutes of decreased intensity activity

3-4 minutes of static stretching

1-2 minutes of relaxation breathing

Adherence Enhancement

Social Support Integration:

Coordinate exercise sessions with husband when possible.

Consider community-based programs for diabetes exercise.

Water aerobics classes provide social interaction and supervision.

Monitoring Tools:

Provide exercise diary with glucose monitoring integration.

Consider activity tracker to monitor steps and activity patterns.

Weekly self-assessment of progress toward goals

Behavioral Strategies:

Schedule exercise at consistent times

Identify specific barriers and develop contingency plans.

Use "habit stacking" to pair exercise with established daily routines.

Lifestyle Integration

Workplace Adaptations:

Set hourly reminders to break up sitting time.

Perform seated stretches during work breaks.

Use lunch break for short walking session.

Home Environment:

Designate specific areas for exercise equipment

Schedule TV time with concurrent stationary cycling

Place visual reminders of exercise plan in prominent locations

**EVALUATION AND FOLLOW-UP**

Strength and endurance measures

Flexibility assessment

Blood glucose control patterns

Body composition changes

Quality of life measures

**12**

**Figure**

**
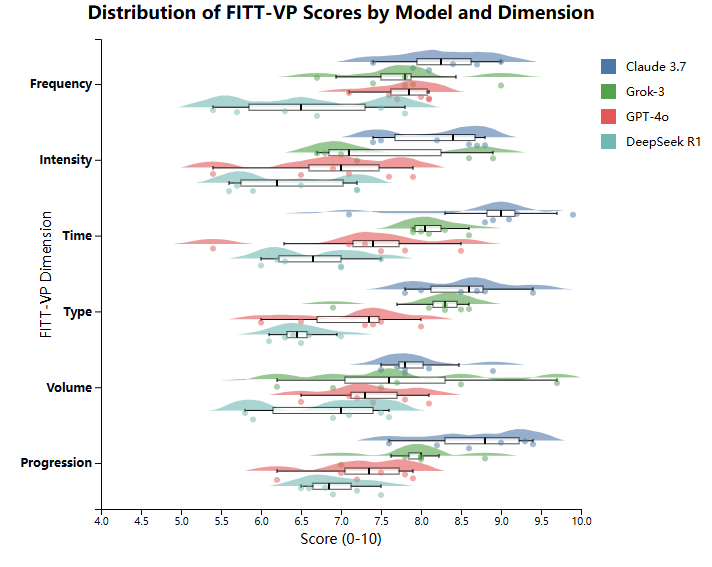
**

**Figure S2 Distribution of FITT-VP Scores by Model and Dimension.**

**
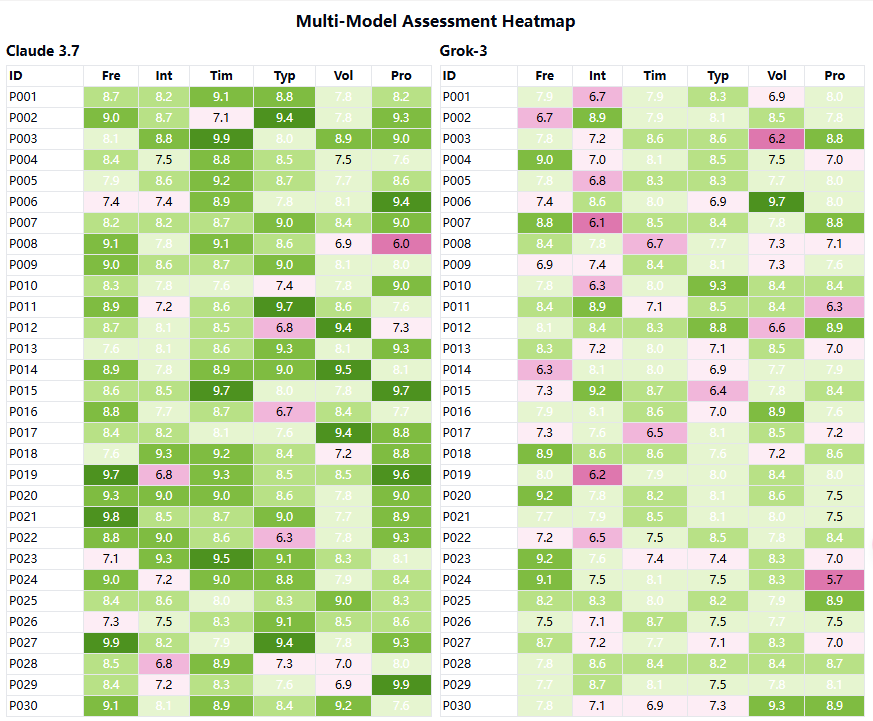
**

**
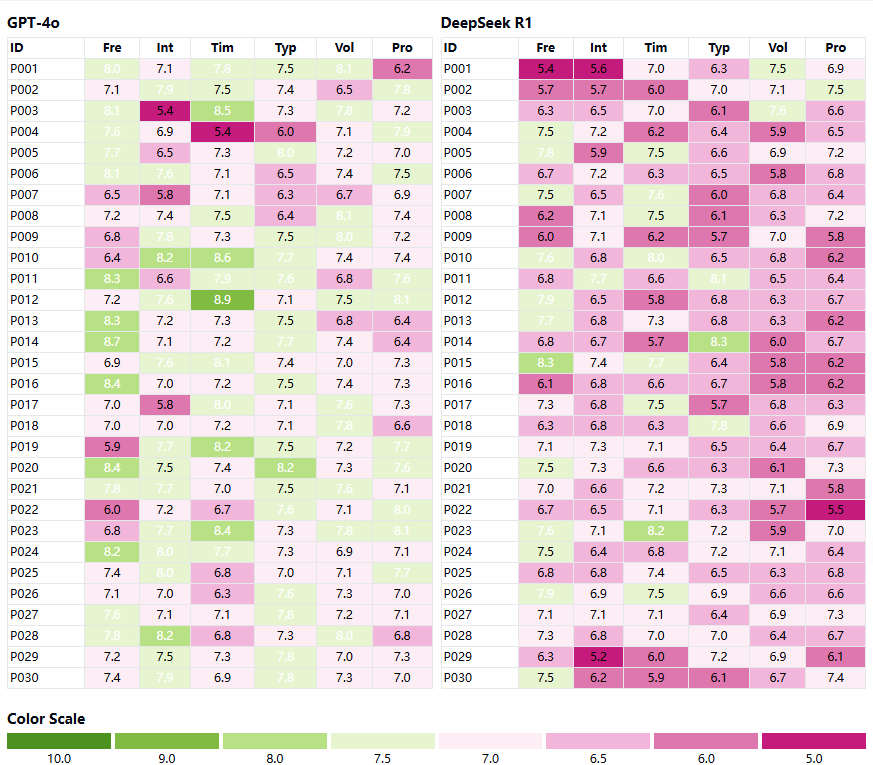
**

**Figure S3 for model performance heatmap**
